# Relationship Between Theory of Mind, Emotion Recognition, and Social Synchrony in Adolescents With and Without Autism

**DOI:** 10.3389/fpsyg.2018.01337

**Published:** 2018-07-31

**Authors:** Paula Fitzpatrick, Jean A. Frazier, David Cochran, Teresa Mitchell, Caitlin Coleman, R. C. Schmidt

**Affiliations:** ^1^Department of Psychology, Assumption College, Worcester, MA, United States; ^2^Department of Psychiatry, University of Massachusetts Medical School, Worcester, MA, United States; ^3^Department of Psychology, Brandeis University, Boston, MA, United States; ^4^Department of Psychology, College of the Holy Cross, Worcester, MA, United States

**Keywords:** autism, theory of mind, emotion recognition, social synchrony, social motor skill

## Abstract

Difficulty in social communication and interaction is a primary diagnostic feature of ASD. Research has found that adolescents with ASD display various impairments in social behavior such as theory of mind (ToM), emotion recognition, and social synchrony. However, not much is known about the relationships among these dimensions of social behavior. Adolescents with and without ASD participated in the study. ToM ability was measured by viewing social animations of geometric shapes, recognition of facial emotions was measured by viewing pictures of faces, and synchrony ability was measured with a spontaneously arising interpersonal movement task completed with a caregiver and an intentional interpersonal task. Attention and social responsiveness were measured using parent reports. We then examined the relationship between ToM, emotion recognition, clinical measures of attention and social responsiveness, and social synchronization that arises either spontaneously or intentionally. Results indicate that spontaneous synchrony was related to ToM and intentional synchrony was related to clinical measures of attention and social responsiveness. Facial emotion recognition was not related to either ToM or social synchrony. Our findings highlight the importance of biological motion perception and production and attention for more fully understanding the social behavior characteristic of ASD. The findings suggest that the processes underlying difficulties in spontaneous synchrony in ASD are different than the processes underlying difficulties in intentional synchronization.

## Introduction

Impairments in social interaction and communication and the presence of restricted and repetitive behaviors (RRBs) are core features of autism spectrum disorder (ASD, American Psychiatric Association [APA], 2013) but the specific processes underlying the disorder are not well understood. Despite extensive research that has uncovered fundamental problems in development of theory of mind (ToM, Baron-Cohen et al., 1985; Happé, 1993; Peterson et al., 2012; Mathersul et al., 2013; Wellman and Peterson, 2013; Kimbi, 2014), motor skills (Ghaziuddin and Butler, 1998; Pan et al., 2009; Fournier et al., 2010; May et al., 2016; Wilson et al., 2018), and emotion recognition (Hobson, 1986; Celani et al., 1999) in individuals with ASD, no one theory has been able to fully account for all features of the disorder. In addition, much of the research reveals a mix of both deficits and competencies in these abilities (e.g., Wellman et al., 2001; Harms et al., 2010; Gonzalez-Gadea et al., 2013; Hutchins et al., 2016), perhaps a result of the heterogeneity inherent across individuals. Part of the problem may be due to reliance on methodological approaches that are not sensitive enough for understanding complex, dynamic social interactions that unfold over time. Furthermore, the role of rhythmicity and social synchrony, which have been shown to increase feelings of rapport, affiliation, and cooperation (Hove and Risen, 2009; Miles et al., 2009; Wiltermuth and Heath, 2009), are under-explored areas in ASD research. Research is needed that not only presents stimuli and measures behavior dynamically in real time during social exchanges but also evaluates the relationship among the clinical, social cognitive, and synchrony variables.

Certainly, responding to social stimuli is a dynamic process that changes from moment to moment during everyday interactions. This sort of fluid exchange allows for not only smoother interactions between people, but also for a mutual understanding of the goals and perspectives of each party involved. Therefore, conducting research in ways (a) to mimic the fact that social tasks change in very complex and unpredictable ways, and (b) to measure continuous ‘on-line’ adjustment of behavior may provide new avenues for better understanding the social behavior of those with ASD, especially the types of social adaptation that is particularly problematic in more naturalistic social situations. Below, we review research findings regarding ToM, emotion recognition, and social synchrony in ASD that provide the backdrop for the current research. We highlight ToM research employing depictions of animated shapes (Abell et al., 2000; Klin, 2000) that demonstrates the value of using dynamic stimuli, and social synchronization research from a behavioral dynamics perspective (Kelso, 1995) that illustrates the value of time-series measures of social behavior.

### ToM and ASD

One important cognitive ability that contributes to social interactions and communication is ToM. ToM refers to the ability to make judgments about another person’s mental state on the basis of behavioral observation. This allows for a mutual understanding that each individual has their own set of beliefs, desires, and intentions that can be applied to create a meaningful social interaction (Meltzoff and Gopnik, 1994). There is a substantial body of research evidence that a ToM deficit or ToM developmental delay is a fundamental feature of ASD (Baron-Cohen et al., 1985; Meltzoff and Gopnik, 1994; Reed, 1994; Baron-Cohen, 1995; Peterson, 2002; Hamilton et al., 2007; Peterson et al., 2012; Mathersul et al., 2013; Wellman and Peterson, 2013; Kimbi, 2014). However, while many children and adults with ASD have been found to perform poorly on verbal ToM tasks (Baron-Cohen et al., 1985; Reed, 1994; Hamilton et al., 2007), children have been found to be equivalent to their typically developing peers in emulating the intended actions of others (Carpenter et al., 2001), and in helping tasks (Liebal et al., 2008), suggesting perhaps those with ASD can make inferences about another’s mental state. In addition, some higher functioning individuals with ASD and Aspergers disorder have been reported to succeed at ToM tasks (e.g., Bowler, 1992). More recently, some researchers have disputed whether the social communication and interaction problems evident in ASD stem from a core impairment in ToM (e.g., Chevallier et al., 2012; Dufour et al., 2013; Scheeren et al., 2013).

Frith and Happé (1994) have argued that the ToM account of autism does not fully explain all the features of the disorder or the heterogeneity across individuals (i.e., why some individuals are able to succeed at ToM tasks) and have proposed that difficulties in central coherence (the ability to integrate information across local and global levels) may be a useful framework for understanding ASD. On the other hand, in his 15-year review of ToM research, Baron-Cohen (2001) concluded that a ToM deficit is a core and universal feature of ASD. He argued that in spite of isolated instances whereby some children and adolescents are able to pass some ToM tasks, there is always a significant delay in the development of ToM and difficulty in performing higher-order ToM tasks persists. Similarly, other researchers have argued that given the incredible heterogeneity evident in ASD, especially with respect to executive functioning, it is not surprising that isolated pockets of competency can be found even amidst the rather pervasive ToM deficits (Gonzalez-Gadea et al., 2013; Hutchins et al., 2016). Other research, which has demonstrated the use of compensatory strategies in some individuals with ASD (Livingston et al., 2018), provides another framework for understanding some of the apparent inconsistencies in the literature.

Still other researchers have questioned the sensitivity of some measures of ToM to adequately assess the type of mentalizing needed in daily life. For example, Abell et al. (2000) and Klin (2000) have suggested that traditional ToM tasks may not be sufficient measures of the type of social adaptation skills needed in naturalistic social interactions. Instead, they had participants complete a social attribution task after viewing animations of geometric shapes based on Heider and Simmel (1944) films to mimic the more implicit and dynamic processing required in social interactions. Abell et al. (2000) found that high functioning children with ASD made inaccurate mental attributions, even if they successfully passed a traditional ToM task. Similarly, Klin (2000) found that high functioning individuals with autism and Aspergers disorder performed poorly on the animations task despite successful completion of a traditional ToM task. This task may be more sensitive than other types of ToM tasks because it is a task that unfolds over time and requires “on-line mentalizing” and thus makes the use of compensatory mechanisms less likely. White et al. (2011) extended work regarding the animations task and developed an objective, multiple-choice tool for assessing ToM. They found it to be equivalent to subjective measures and recommended its adoption for children and adults due to the fact that it is faster to administer and more objective.

Other research (Gobbini et al., 2007) found evidence that more cognitive assessments of ToM (e.g., traditional false belief tasks) activate different brain regions than tasks in which intentional attributions are made on the basis of perceived actions (e.g., animations of geometric shapes), suggesting that the attribution of agency and mental states may not involve a singular neural system. The false belief tasks activated the anterior paracingulate cortex (APC), the posterior cingulate cortex/precuneus (PCC/PC), and the temporo-parietal junction (TPJ) while the social animation tasks activated the posterior superior temporal sulcus (pSTS), as well as the frontal operculum and inferior parietal lobule (IPL). This interpretation was also confirmed by Zwickel et al. (2011) behavioral data which revealed that those with ASD who had some intact foundational social processing (perceiving agency of, and adopting the visuo-spatial perspective of another) nevertheless were not able to attribute mental states to others on the basis of that information. Kimbi’s (2014) review of the literature similarly revealed that performance was worse on implicit ToM tasks compared to explicit tasks. Thus, it seems likely that the discrepancies in ToM performance may be due to the attribution of mental states involving different underlying mechanisms than perceiving agency and adopting visuo-spatial perspective. Some individuals with ASD may have intact mechanisms for perceiving agency and visuo-spatial perspective but have dysfunctional mechanisms responsible for the attribution of agency.

### Emotion Recognition and ASD

The ability to recognize and interpret the emotions of other is another important dimension of social interactions and communications. A number of research studies have reported that the ability to recognize the emotions of others is disrupted in ASD (e.g., Baron-Cohen et al., 1985; Hobson, 1986; Bölte and Poustka, 2003; Kuuskikko et al., 2009) but others have reported that emotion recognition was not impaired (Prior et al., 1990; Capps et al., 1992; Robel et al., 2004; Castelli, 2005). Harms et al. (2010) reviewed the literature on facial emotion recognition (FER) and suggest that the mixed research findings regarding the existence of FER deficits in ASD can be explained by three factors —demographic characteristics of the participants, task demands, and the specific behaviors measured. In addition, they concluded that some evidence suggests that the mechanisms used for identifying facial expressions is different in those with ASD than without. Thus, even though some individuals with ASD may successfully complete some emotion recognition tasks, the process of how they do so is different than those without ASD. This conclusion was confirmed recently by Black et al.’s (2017) review of the literature on emotion recognition in eye tracking and electroencephalography (EEG) studies where the authors concluded that visual processing of emotion is different in those with ASD than without.

### Social Synchrony and ASD

A less researched component of social interaction and communication in ASD is associated with social motor processes. This involves the coordination of body movements during social interaction that become synchronized over time. Bernieri et al. (1994) found that this sort of social synchrony is an essential part of successful interactions because it is a vital feature of interpersonal responsiveness, social rapport, and other directedness. Some research evidence indicates that social synchrony breaks down in ASD. For example, synchronization of speech and gesture was found to be disrupted in ASD (de Marchena and Eigsti, 2010), as was the timing of facial mimicry (Oberman et al., 2009). In addition, Tordjman et al. (2015) proposed that ASD is a disorder of biological and behavioral rhythms and reported that some treatments using melatonin (due to its role in regulating circadian rhythms) and behavioral interventions based on synchrony have had promising preliminary results as treatments for ASD.

It should be pointed out that interpersonal synchronization can arise either intentionally when there is an explicit social goal or spontaneously without conscious awareness when there is no explicit goal (Schmidt et al., 2011). Research from a coordination dynamics approach has expanded our understanding of the relevance of social synchrony in ASD through its recording of continuous time-varying measures of behavior as it unfolds and using time-series analysis techniques to analyze the patterning, structure, and strength of system components as they change over time. Research using this methodology has found that school-age children with ASD had lower social motor synchronization abilities than controls (Fitzpatrick et al., 2013, 2017a). In addition, Marsh et al. (2013) found that preschoolers with ASD spontaneously synchronized their rocking chair movements with a partner less often than controls.

Similarly, Fitzpatrick et al. (2016) found that adolescents with ASD demonstrate less synchronization in both spontaneous and intentional interpersonal coordination than adolescents without ASD. This research is particularly noteworthy because using a simple interpersonal task, namely, the rhythmic coordination of hand-held pendulums, the researchers were able to employ direct dynamical modeling that has been used to differentiate other populations with social deficits. This task not only revealed that dynamical models of intentional and spontaneous motor synchronization could differentiate adolescents with and without ASD, but findings also suggest that joint disruptions in both spontaneous and intentional synchronization may be a unique feature of the social difficulties in ASD. Research on individuals with schizophrenia, for example, found spontaneous synchronization was equivalent for individuals with and without schizophrenia, but intentional synchronization was lower for those with schizophrenia (Varlet et al., 2012). Del-Monte et al. (2013) found a similar pattern of results with first-degree relatives of individuals with schizophrenia.

### Research Questions

As indicated above, the ability to attribute mental states based on viewing another person’s behavior, the ability to recognize and understand the emotions displayed in facial expressions, and the ability to engage in spontaneous and intentional synchronization of body movements all play important roles in social interaction and communication. There is evidence that all three of these dimensions of social action-based behavior appear to be disrupted in ASD. However, the relationships among these action-based behaviors are not well understood and the main focus of this paper is to examine the relationship among these variables. A lack of significant relationships among the variables could indicate that ToM, emotion recognition, and social synchrony have independent underlying processes. Alternatively, significant relationships between measures could indicate that they either share underlying processes or a disruption in one may lead to a disruption in another.

Previous research provides the basis for evaluation of more specific predictions. With respect to ToM and emotion recognition, Campbell et al. (2006) found that facial emotion recognition and ToM were not correlated in typically developing children, although perception of intention based on gaze was correlated with ToM. They concluded that gaze processing and ToM may share similar neural circuitry, but ToM and emotion recognition are dissociable in typical development. While they found poorer performance in both an animations ToM task and facial emotion recognition task, they did not evaluate the relations between face-processing and ToM abilities in the adolescents with ASD. If ToM and emotion recognition abilities represent independent developmental continuums, then one would expect that emotion recognition and ToM should not be correlated in our sample.

In terms of the relationship between synchrony and ToM, Fitzpatrick et al. (2017b) found that social synchrony in children was related to performance on false-belief ToM tasks, suggesting these tasks may share underlying processes. Of interest here is whether social synchrony and animation ToM tasks demonstrate a similar relationship. In addition, our methodology provides a more stringent evaluation of whether the relationship between synchrony and ToM is different for spontaneous and intentional synchronization. The relationship between social synchrony and facial emotion recognition has not been previously evaluated. If ToM and social synchrony share underlying circuitry, one would expect that synchrony would not be related to facial emotion recognition.

In addition, we compared performance of adolescents with and without ASD on a ToM animations task and emotion recognition task, and expected to replicate previous research findings of Campbell et al. (2006). Namely, we expected that adolescents with ASD would be less able to accurately make mental attributions of animated movements or recognize facial emotions.

## Materials and Methods

### Participants

The participants consisted of 18 adolescents paired with a parent. Nine adolescents had a diagnosis of autism (8 males, 1 female, average age 13.67 years, range 12–17) and nine were neuro-typical (7 males, 2 females, average age 14.44 years, range 12–16). The participants were matched for chronological age.

The Wechsler Abbreviated Scale of Intelligence (WASI; Wechsler, 2011) was used to ensure subjects met the intelligence requirement for inclusion. The WASI is a short reliable measure of intelligence and is nationally standardized and we used the matrix reasoning and vocabulary subtests. Intelligence was in the normal range (85–115) for both groups but the WASI score of the ASD group was slightly lower than the control group (see **Table 1**).

**Table 1:** Participant characteristics and clinical phenotyping.

	ASD (*n* = 9)		Neuro-typical (*n* = 9)		Group difference	
	Mean	*SD*	Mean	*SD*	*t*(16)	*p*
CA (years)	13.67	1.94	14.44	1.13	-1.04	0.31
WASI vocabulary	52	10.99	63.78	6.72	-2.74	0.01
WASI matrix	49.78	7.12	55.44	8.75	-1.51	0.15
WASI IQ	101.78	13.84	117.22	13.15	-2.43	0.03
ADOS					
Communication	3.11	0.93	0.22	0.44	8.44	<0.001
Social interaction	5.44	2.19	0.11	0.33	7.24	<0.001
Communication and social interaction total	8.56	2.92	0.33	0.5	8.33	<0.001
Stereotyped behaviors and restricted interests	2.0	1.41	0	0	4.26	0.001
SRS (*t*-score)	77.0	17.65	38.33	2.96	6.48	<0.001
ADHD inattention	285.70	29.68	276.14	19.43	0.81	0.43
ADHD hyperactivity	203.28	45.34	109.19	11.92	6.02	<0.001
ADHD total	488.99	67.91	385.33	28.81	10.46	0.001
SCQ	17.78	9.54	1.44	1.24	5.10	<0.001
CBCL					
Social problems	5.78	3.99	0.67	0.71	3.78	0.002
Thought problems	6.89	2.15	0.11	0.33	9.34	<0.001
Attention problems	8.89	4.23	1.00	1.12	5.34	<0.001
Social relations	6.78	3.36	10.89	2.75	-2.84	0.01
ADHD	6.44	3.21	0.67	1.00	5.16	<0.001

Pre-screening procedures were used to ensure that participants with ASD had been previously diagnosed with ASD by a licensed clinical psychologist or medical doctor based on DSM-IV_TR criteria (American Psychiatric Association [APA], 2000). The Autism Diagnostic Observation Schedule, Second Edition (ADOS-2; Lord et al., 2012) was administered to all participants to confirm diagnostic group membership. The ADOS is a semi-structured, standardized assessment of communication, social interaction, and play for individuals referred because of possible autism. It is comprised of five modules – a toddler module for children between 12 and 30 months of age, and four modules appropriate for use with individuals of varying language levels and age; these range from Module 1, intended for young children who are non-verbal or using mostly single words, Module 2, intended for children of any age who use phrase speech, Module 3, intended for verbally fluent children and young adolescents, and Module 4, intended for verbally fluent older adolescents and adults. The ADOS subscores for communication, social interaction, and stereotyped behaviors and restricted interests, as well as the communication and social interaction total, are reported. Five participants were administered Module 3 and 13 were administered Module 4. The mean ADOS scores for the ASD group was significantly higher than the neuro-typical group and confirmed group membership (see **Table 1**).

Parents provided written, informed consent for children to participate, parents signed releases to consent to video recordings, and adolescents gave their assent to participate. The study was conducted over two visits and the first visit took approximately 3 h while the second visit took approximately 1.5 h. Participants received $50 for the first visit and $50 for the second visit. The project was approved by the University of Massachusetts Medical School Institutional Review Board (IRB) and the Assumption College IRB. Participants were recruited from local communities through print advertising, a recruitment brochure, email, social media, and community events. Recruitment material was posted on various community and University of Massachusetts Medical School websites.

### Clinical and Social Measures

There were two separate experimental sessions, completed approximately 1-week apart. In the first session, screening for medical and psychopathology was conducted. The appropriate ADOS module based on participant language and developmental level was administered, as were the WASI Matrix Reasoning and Vocabulary Subtests. Both the ASD group and control group completed the ADOS. The Social Responsiveness Scale (SRS; Constantino and Gruber, 2005), Attention Deficit/Hyperactivity Disorder (ADHD) Rating Scale IV (DuPaul et al., 1998), Child Behavior Checklist (CBCL; Achenbach and Rescorla, 2001), and Social Communication Questionnaire (SCQ: Rutter et al., 2003) were completed by the parent. Each of these measures is described below.

#### Social Responsiveness Scale

The Social Responsiveness Scale (SRS; Constantino and Gruber, 2005) is a 65-item rating scale that measures the severity of autism spectrum symptoms as they occur in natural social settings. It is appropriate for use with children from 4 to 18 years of age. The SRS is completed by a parent in 15 to 20 min. It provides a clear picture of a child’s social impairments, assessing social awareness, social information processing, capacity for reciprocal social communication, social anxiety/avoidance, and autistic preoccupations and traits. We used the SRS *t*-score in the current study.

#### ADHD Rating Scale IV

The ADHD Rating Scale IV (DuPaul et al., 1998) assesses symptoms of inattention, hyperactivity, and impulsivity and is based on the DSM diagnostic criteria for attention deficit hyperactivity disorder. The ADHD inattention, hyperactivity, and total scores were used in the data analysis.

#### Child Behavior Checklist

The Child Behavior Checklist (CBCL; Achenbach and Rescorla, 2001) is a parent-report questionnaire that provides a measure of behavioral and emotional problems. In this paper, the following subscales, related to ASD traits, were used: social problems, thought problems, attention problems, social relations, and ADHD.

#### Social Communication Questionnaire

The Social Communication Questionnaire (SCQ: Rutter et al., 2003) is a brief parent-report instrument to evaluate communication skills and social functioning. It is appropriate for use in children 4 years or older. The overall SCQ summary scores was used in the data analysis.

### Experimental Tasks and Procedure

In the second visit, three experimental tasks (social synchronization, ToM, and recognition of facial emotions) were completed. The spontaneous synchrony task was completed at the start of the experimental session to prevent experimental task demands from influencing performance and the order of presentation of the in-phase and anti-phase intentional synchrony trials was counterbalanced across participants. Presentation software (Neurobehavioral Systems Inc., Berkeley, CA, United States) running on a Dell computer was used to present the stimuli for the ToM and emotional recognition tasks.

The data collected for this study was part of a larger project investigating differences in spontaneous and intentional synchronization abilities of adolescents with and without ASD (Fitzpatrick et al., 2016). Here, we used three of the measures from Fitzpatrick et al. (2016) as an index of synchronization ability (circular variance for spontaneous, intentional in-phase, and intentional anti-phase coordination, described below) and analyzed the relationship between synchronization and clinical psychopathology, and social skills measures (parental report and experimental) not previously reported.

### Experimental Tasks

#### Social Synchronization Tasks

The social synchronization tasks involved adolescent-parent pairs swinging hand-held pendulums while the movement time-series of the pendulums was recorded. During the spontaneous synchrony task, participants looked at their partner’s pendulum but were instructed to swing at their own comfortable tempo and maintain that tempo throughout the trial. During the intentional synchrony task, participant pairs coordinated their pendulum swinging with their partner in either an in-phase pattern (so their pendulums were in the same portion of their cycles at the same time) or anti-phase pattern (so that their pendulums were in opposite portions of their cycles at the same time). Additional details about the task and calculation of the synchrony measure (circular phase variance) can be found in Fitzpatrick et al. (2016).

#### ToM Task

ToM was measured by having participants view Frith-Happé animations of geometric shapes depicting ToM (social) animations, goal-directed movements, or random movements. The 12 animations originally used in Abell et al.’s (2000) study were the stimuli, with four animations of each type. Responses were measured with the objective multiple-choice test developed by White et al. (2011) in which participants chose one of three categories to rate the interaction in the film clip—no interaction, physical interaction, mental interaction. The maximum score for each animation type was 4. After the ToM animations, participants were presented with two additional questions (MCQ Feelings) to choose the adjective that best described the feelings of the small and large triangles. The maximum score was 8, corresponding to two possible correct answers for each of the 4 animations. The percent correct was calculated for the four ratings (goal-directed, random, ToM, MCQ Feelings) and used in data analysis.

#### Facial Emotion Recognition Task

Recognition of facial emotions was measured using a modified Ekman 60 faces test (Ekman and Friesen, 1976), especially designed for use in individuals with ASD. The participant was instructed to watch a presentation of the stimuli from four picture conditions (angry faces, fearful faces, neutral faces, and houses), all presented in an oval cutout. A blocked design paradigm, with eight items per block (1 repetition, somewhere at random within block) was used. Participants were asked to respond by pressing the space bar whenever the item was repeated—there was always one per block. Blocks alternated between ANGER, FEAR, NEUTRAL, HOUSE, and REST, with a rest block separating all task blocks. Each active block was 20 s, each rest block was 16 s, and there were two blocks per condition across three runs. Reaction time was recorded and the number of hits was calculated for the four picture conditions (anger, fear, neutral faces, and houses).

### Data Analysis

Diagnostic group (ASD, neuro-typical control) was a between-subjects variable. Group differences in clinical and social measures (ADOS, ADHD Rating Scale, SRS, SCQ, CBCL) were evaluated with independent samples *t*-tests. Group differences in ToM scores was analyzed with a 2 (diagnostic group; ASD, neuro-typical control) × 4 (animations rating condition, goal-directed, random, ToM, MCQ Feelings) mixed ANOVA. Diagnostic group was a between-subjects variable and animations rating condition was a within-subjects variable. Group differences in facial emotion recognition was analyzed with a 2 (diagnostic group) × 4 (picture conditions (anger, fear, neutral faces, and houses) mixed ANOVA. Diagnostic group was a between-subjects variable and picture condition was a within-subjects variable.

In order to evaluate the relationship between spontaneous synchronization and clinical and social measures (ADOS, WAIS, ADHD Rating Scale, SRS, SCQ, CBCL, ToM, and emotion recognition), bivariate correlations with circular variance during the spontaneous coordination condition (i.e., the looking condition) were conducted. Data from both groups (adolescents with and without ASD) were included in these analyses to explore the relationships evident across a broader range of skills. In addition, an exploratory factor analysis was conducted to further explore the underlying relationships between these variables.

## Results

### Group Differences in Clinical Phenotyping

As seen in **Table 1**, independent samples *t*-tests revealed significant group differences for all the clinical phenotyping measures except for the ADHD Inattention scale. SRS, ADHD Hyperactivity, SCQ, CBCL Thought Problems, CBCL Attention Problems, and CBCL ADHD had the most significant differences between groups.

### Are There Group Differences in ToM?

**FIGURE 1:**
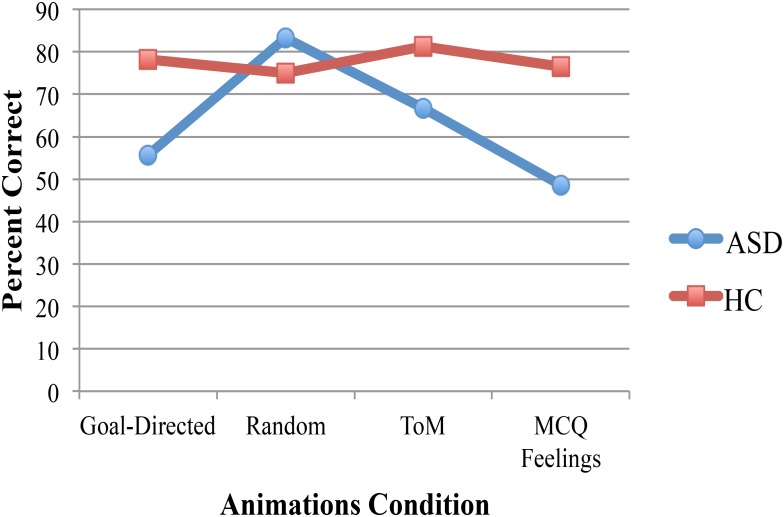
Significant group differences in performance on the animations task was due largely to significantly worse performance by adolescents in the ASD group on the MCQ Feelings condition than control participants, no significant group differences were found for the other animations conditions. Participants in the ASD group were significantly better in the random movements condition than the MCQ Feelings condition but the performance of control participants was equivalent for all animations conditions.

A 2 (diagnosis group) × 4 (animations rating condition) mixed ANOVA with a dependent variable of percent correct resulted in a significant main effect of diagnosis group [*F*(1,15) = 4.6, *p* = 0.05, η^2^ = 0.24]. Participants with ASD had lower scores (*M* = 63.45) than controls (*M* = 77.73). Neither the animations condition nor the interaction was significant. Bonferroni *post hoc* comparisons that investigated the simple effects of the interaction revealed that the group difference was largely accounted for by a significant group difference on MCQ Feelings (*p* = 0.04, *M*_Difference_ = -27.42, see **Figure 1**). The group difference for goal directed movements approached significance (*p* = 0.08, *M*_Difference_ = -22.57) but neither random nor ToM resulted in significant group differences (*p* = 0.56 and *p* = 0.11 for random and ToM, respectively). In addition, for the ASD group, random was significantly different from MCQ Feelings (*p* = 0.05, *M*_Difference_ = 34.72) but no other animation conditions were significantly different from each other. For the control group, there were no significant differences in performance on any of the animation conditions.

### Are There Group Differences in Facial Emotion Recognition?

**FIGURE 2:**
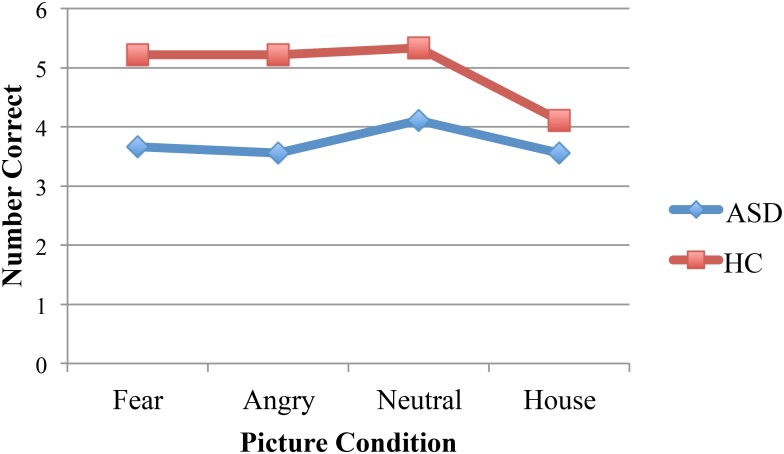
Participants with ASD performed worse than the control participants in all three picture conditions with faces. Performance was equivalent between groups for the pictures of houses.

A 2 (diagnosis group) × 4 (picture condition) mixed ANOVA with a dependent variable of number correct resulted in a significant main effect of diagnosis group [*F*(1,16) = 9.19, *p* = 0.008, η^2^ = 0.37]. ASD participants demonstrated less facial recognition (*M* = 3.72) than the control group (*M* = 4.97). The main effect of task approached significance [*F*(3,48) = 2.38, *p* = 0.08, η^2^ = 0.13] and the interaction was not significant. Bonferroni *post hoc* comparisons that investigated the simple effects of the interaction revealed significant group differences for all three faces conditions (*p* = 0.02, *p* = 0.008, *p* = 0.03, for the fear, angry, and neutral faces) but not for the houses (*p* = 0.43, see **Figure 2**). A 2 (diagnosis group) × 4 (picture condition) ANOVA with a dependent variable of reaction time did not result in any significant main effects for diagnosis group [*F*(1,16) = 2.65, *p* = 0.12, η^2^ = 0.14] or task [*F*(3,48) = 1.63, *p* = 0.20, η^2^ = 0.09]. The interaction was also not significant [*F*(3,48) = 0.06, *p* = 0.98, η^2^ = 0.004].

### Relationship Between ToM and Emotion Recognition

The correlations between the two ToM tasks (ToM animations, MCG Feelings) and emotion recognition tasks (Fear and Angry displays) were of interest. As seen it **Table 2**, none of these correlations were significant. There was one significant correlation between one ToM control task (GD ToM) and one control emotion recognition task (neutral face).

**Table 2:** Correlations (*N* = 18) between ToM (percent correct) and emotion recognition (hits).

	Emotion recognition			
	Fear	Angry	Neutral	Houses
TOM				
GD	0.26	0.04	0.57*	-0.07
Random	-0.34	-0.39	-0.005	-0.25
ToM	0.19	0.31	0.44	0.34
MCQ feelings	0.35	0.27	0.36	0.07

*^∗^p = 0.02.*

### Relationship Between Synchronization and Other Measures of Social Functioning

#### Spontaneous Coordination

Circular phase variance during spontaneous entrainment was significantly correlated with WASI Vocabulary and WASI IQ, but there were no other significant correlations with any other clinical measures (see **Table 3**). Circular phase was significantly correlated with two of the ToM conditions, GD and MCQ Feelings, but not with random or ToM. Circular phase variance during spontaneous entrainment was not significantly correlated with any of the emotion recognition tasks.

**Table 3:** Correlations (*N* = 18) between unintentional and intentional synchronization and clinical measures.

	Spontaneous entrainment		Intentional coordination			
			In-phase		Anti-phase	
	*r*	*p*	*r*	*p*	*r*	*p*
WASI vocabulary	0.48	0.04^*^	0.20	0.43	0.18	0.48
WASI matrix	0.39	0.11	0.07	0.77	-0.02	0.95
WASI IQ	0.49	0.04^*^	0.17	0.51	0.12	0.65
ADOS					
Communication	-0.32	0.20	-0.54^*^	0.02	-0.58^**^	0.01
Social interaction	-0.41	0.096	-0.49^*^	0.04	-0.43	0.07
Communication and social interaction total	-0.38	0.12	-0.51^*^	0.03	-0.49^*^	0.04
Stereotyped behaviors and restricted interest	-0.17	0.52	-0.28	0.28	-0.30	0.24
SRS (*t*-score)	-0.21	0.41	-0.62^**^	0.006	-0.65^**^	0.003
ADHD inattention	0.31	0.21	-0.39	0.11	-0.45	0.06
ADHD hyperactivity	-0.13	0.61	-0.61^**^	0.007	-0.68^**^	0.002
ADHD total	0.003	0.99	-0.61^**^	0.007	-0.69^**^	0.002
SCQ	-0.06	0.81	-0.45	0.06	-0.57^**^	0.01
CBCL					
Social problems	-0.13	0.61	-0.69^**^	0.002	-0.67^**^	0.002
Thought problems	-0.32	0.19	-0.58^**^	0.01	-0.65^**^	0.003
Attention problems	-0.22	0.39	-0.54^*^	0.02	-0.61^**^	0.008
Social relations	0.14	0.58	0.68^**^	0.002	0.71^**^	0.001
ADHD	-0.23	.35	-0.82^**^	<0.001	-0.76^**^	<0.001

*^∗^p < 0.05; ^∗∗^p < 0.01.*

#### Intentional Coordination

In order to evaluate the relationship between intentional synchronization and more traditional measures of social skills (clinical scales and experimental measures of ToM and emotion recognition), a composite score of circular phase variance for all three pendulum conditions was calculated for both in-phase and anti-phase. Bivariate correlations were conducted with circular phase in-phase and anti-phase composite scores. As seen in **Table 4**, either intentional in-phase or anti-phase circular phase variance was correlated with WASI Vocabulary, Matrix, or IQ. However, there were significant correlations with the CBCL measures (Social Problems, Thought Problems, Attention Problems, Social Relations, and ADHD) and SRS for both in-phase and anti-phase. In addition, there were significant correlations with the ADOS communication and social interaction subscales, but not the stereotyped behaviors and restricted interests. The correlations were also significant for ADHD hyperactivity and SCQ was correlated with anti-phase synchronization but not in-phase. MCQ Feelings was significantly correlated with in-phase synchronization but no other ToM or emotion recognition correlations were significant.

**Table 4:** Correlations (*N* = 18) between unintentional and intentional synchronization and ToM and emotion recognition.

	Spontaneous entrainment		Intentional Coordination			
			In-phase		Anti-phase	
	*r*	*p*	*r*	*p*	*r*	*p*
ToM animations (percent correct)		
GD	0.50^*^	0.04	0.04	0.88	0.10	0.72
Random	0.02	0.95	-0.16	0.55	-0.14	0.61
ToM	0.23	0.37	0.01	0.96	-0.06	0.83
MCQ feelings	0.63^**^	0.007	0.48^*^	0.05	0.36	0.15
Emotion recognition (hits)						
Fear	0.33	0.18	0.30	0.22	0.18	0.47
Angry	0.18	0.48	0.39	0.11	0.31	0.22
Neutral	0.40	0.10	-0.06	0.81	0.03	0.90
Houses	0.22	0.38	0.08	0.74	0.08	0.76

*^∗^p < 0.05; ^∗∗^p < 0.01.*

### Factor Analysis

In order to determine whether there were latent factors or components underlying the correlations between variables measuring different aspects of social abilities, a principal components factor analysis was conducted. We included variables that measured a range of behaviors that successfully differentiated the groups in the previous analyses—intentional synchronization ability (anti-phase circular phase), unintentional/spontaneous synchronization ability (circular phase during the looking condition), intelligence (WASI IQ), communication ability (ADOS Communication and Social Interaction), attention (CBCL ADHD), social responsiveness (SRS), and ToM (MCQ Feelings). Some behaviors were measured multiple ways so we chose the measure that best differentiated the groups for inclusion in this analysis.

The performed factor analysis satisfied several adequacy criteria. First, all items correlated at least 0.4 with at least one other item, suggesting reasonable factorability. Second, the Kaiser–Meyer–Olkin measure of sampling adequacy was 0.79 (above the recommended value of 0.5), and Bartlett’s test of sphericity was significant [χ^2^(21) = 67.03, *p* < 0.001]. Additionally, the communalities were all above 0.5, confirming that each item shared some common variance with other items.

A principal components factor analysis using varimax (orthogonal) rotation found that the three factors explained 87.26% of the variance. The loadings less than 0.40 were excluded. The results of this solution are shown in **Table 5**. Four items, the intentional synchronization, communication, attention, and social responsiveness loaded onto factor 1 and explained 40.18% of the variance. This factor seems to be indexing social action and attention aspects of social skills. Being able to intentionally coordinate one’s action, communicate effectively, and respond appropriately to others, different dimensions of more explicit social action, may share some underlying similarities and are all related to attention. Two items, ToM and spontaneous synchrony, loaded onto factor 2 that explained and additional 24.08% of the variance. This factor seems to be indexing more implicit social knowledge that arises from viewing actions (in the case of ToM) or performing actions with another person. The ToM task measures how well the individual understands the goals of others and this kind of knowledge similar to what is necessary for smoothly and naturally coordinating one’s movements with another during joint actions. Interestingly, the intentional synchrony measure loaded on the social action factor while spontaneous synchrony loaded on a separate factor, suggesting that intentional and spontaneous synchrony may contribute to social interactions in different ways and perhaps have different underlying mechanisms. The final factor was comprised of three items, IQ, communication, and social responsiveness, and explained an additional 22.99% of the variance. This factor could be characterized as social communication and seems to involve somewhat higher order cognitive abilities. In contrast, social action appears to depend more on attention processes.

**Table 5:** Principal components analysis.

Component	1	2	3
Intentional synchrony anti-phase	-0.92		
Spontaneous synchrony		0.92	
Intelligence			-0.86
ToM	0.76		
Communication ability	0.60		0.66
Attention	0.89		
Social response	0.82		0.44

## Discussion

Overall, our results indicated that adolescents with ASD had difficultly ascribing appropriate feelings to ToM animations, although they were equivalent to controls in their ability to detect that a mental state was represented in the ToM animations. In addition, adolescents with ASD performed worse than the controls on facial emotion recognition in terms of accuracy but their reaction times were similar to controls. Interestingly, we found significant relationships between both spontaneous and intentional synchrony and the ability to accurately ascribe feelings in the ToM animations. Intentional synchrony was also related to autism severity, social responsiveness, and attention. In contrast, facial emotion recognition was not correlated with spontaneous or intentional synchrony, nor was emotion recognition related to ToM performance. Furthermore, the principal components analysis resulted in intentional and spontaneous synchrony loading on different factors, suggesting these two types of synchronous behavior tap into different dimensions of social skill that might have different underlying mechanisms. Our findings suggest intentional synchrony shares mechanisms with attention, social communication, and social responsiveness while spontaneous synchrony shares mechanisms with ToM. Facial emotion recognition appears to be dissociated from both of those aspects of social behavior.

Our findings replicated Campbell et al. (2006) in that we also demonstrated that adolescents with ASD have difficulty in perceiving feelings based on movement and recognizing emotion in facial expressions. We also demonstrated that in our sample of adolescents with and without autism, there was no relationship between ToM and emotion recognition. This is consistent with other research (Shamay-Tsoory et al., 2009; Mazza et al., 2014; Baron-Cohen et al., 2015; Pino et al., 2016) that has proposed that the cognitive component of ToM (understanding the mental state of others) and the emotional component (making inferences about someone’s emotional state) may not involve a singular process. Of interest is whether a different sort of ToM task, such as the reading the mind in the eyes test (Baron-Cohen et al., 1997, 2001), would display the same pattern of results. The reading the mind in the eyes test has been extensively researched and found to be a reliable measure of ToM (Fernández-Abascal et al., 2013; Vellante et al., 2013) for both males and females with ASD (Baron-Cohen et al., 2015). It is also possible that the static nature of the facial emotion recognition task lacked the resolution necessary to uncover potentially subtle relationships. Additional research is needed to further explore these alternatives.

Facial emotion recognition was also not related to social synchrony. The lack of a relationship between facial emotion recognition and social synchrony could implicate the ‘online’ processing that is required in tasks that change continuously over time. That is, the emotion recognition task involved a static display, as mentioned above, and both synchronization tasks and the ToM task involved dynamic tasks that were changing over time. Of interest is whether the dynamic, time-dependent processing that was required in those tasks is the salient issue for understanding the social challenges experienced by those with ASD. However, more research is needed to verify that the same pattern emerges for emotional recognition tasks that utilize dynamic displays rather than the static facial displays used here. It could be that processing social information as it unfolds over time utilizes a different set of mechanisms than processing social information from static displays. Alternatively, it could be that facial emotion recognition involves neural processing that is distinct from the processing necessary for attributing mental states or synchronizing one’s movements with another person spontaneously or during joint tasks.

The relevance of motion perception for both making social judgments (as in the case of ToM) and physically engaging in social interactions (as in spontaneous synchronization) is highlighted by the correlations between ToM and spontaneous synchronization and the principal components analysis loading those items together. This finding is also consistent with other research that found that individuals with ASD have trouble detecting aspects of biological motion in point-light displays. For example, individuals with ASD demonstrate deficits in their ability to recognize both emotion and motion as presented in point-light displays (Nackaerts et al., 2012) and a link between social cognition and the perception of biological motion that may be dependent on the severity of ASD (Nackaerts et al., 2012; Miller and Saygin, 2013) has been proposed. Adolescents with autism have also demonstrated the inability to identify the directional movement of a point-light walker performing a consistent motion, leading to the conclusion that there is an overall deficit of perception of biological motion in ASD (Pavlova, 2012). Perhaps the deficit in perception of biological motion is the fundamental problem that results in difficulties in ToM tasks as well as engaging in social synchrony. This raises interesting possibilities for further research to explore if there is common neural circuitry underlying all the tasks.

Also noteworthy is the finding that perception of feelings in the ToM animations was related to spontaneous synchrony but not intentional synchrony. This is consistent with Fitzpatrick et al. (2017b) who also found ToM in children was related to spontaneous synchrony. In contrast, intentional synchronization loaded with communication, social responsiveness, and attention. The correlational nature of this research does not allow us to determine whether one of these factors is driving the disruptions in the others. It might be the case, for example, that the social problems in intentional synchronization, communication, and social responsiveness are actually the result of a general attention processing impairment (Hayes, 1987; O’Riordan et al., 2001), a problem with attention switching (Landry and Bryson, 2004), or an inability of social stimuli to capture attention (Klin et al., 2003; Schultz, 2005; Chawarska et al., 2010; Fischer et al., 2014). Alternatively, it could be the case that there is some other underlying mechanism responsible for the difficulties in synchronization, attention, communication, and social responsiveness. Additional research is needed to disentangle these alternatives.

Nevertheless, the finding that spontaneous and intentional synchrony are related to and load with different social variables raises the interesting possibility that intentional coordination involves different social processes and underlying neural circuitry than spontaneous coordination. Intentional synchrony may be related to the more cognitive component of social understanding as proposed by Baron-Cohen et al. (2015) and others (Shamay-Tsoory et al., 2009; Mazza et al., 2014; Pino et al., 2016) and implicates attention as a variable for further exploration. Our findings that spontaneous synchrony and ToM were not related to emotion recognition suggest that perhaps there is another social processing module that is related to biological motion perception and production. This could mean that in addition to cognitive and emotional components as previously proposed, perhaps there is a social movement component as well.

Identification of a singular mechanism that is able to fully account for all the features of ASD as well as the heterogeneity across individuals has been elusive. The pattern of interrelationships demonstrated in our data is consistent with a more modular account of social behavior characteristic of ASD that involves cognitive, emotional, and social movement components that should be investigated in future research. If the different social processing modules are dissociable, it is possible that an individual has disruptions in one set of processes but not the others. This seems likely given the range of social and communication abilities demonstrated by those with ASD across the spectrum. We speculate that the addition of a social movement component to understanding ASD not only could help explain some of the assets as well as difficulties in social communication and interaction, but also could provide a link for helping to explain the other main diagnostic feature of ASD, the production of RRBs. For example, some researchers have found that RRBs in ASD may function to create rhythmicity, which is sometimes disrupted in individuals with ASD (Tordjman et al., 2015). In addition, some initial evidence (Lampi et al., 2016) has demonstrated that high levels of RRBs were associated with poorer synchronization ability in children, and this relationship remained significant even after controlling for non-verbal ability. We propose that a link between RRBs and social synchrony provides a potential pathway for understanding the two diagnostic features of RRBs and future research is needed to explore this possibility.

### Limitations

Several limitations of this research should be acknowledged. First, these findings are preliminary since this is one of the first studies to examine social synchrony in adolescents. Additional research is needed utilizing larger sample sizes with a wider range of social (synchrony, communication, and social responsiveness), cognitive, and emotional abilities to more fully understand the continuum of ASD traits and relationships among them. In addition, ASD diagnosis was comorbid with attention problems in our sample. Future research is needed to evaluate the relationships among social, cognitive, and emotional abilities in both adolescents with and without ASD who have attention problems as well as those who do not. Finally, social interactions, especially the sorts of physical interactions found in social synchronization, are the result of reciprocal interactions between people. Future research is needed to investigate the reciprocity of coupling between the adolescent and their partner to more fully understand the contribution each makes to the observed behavior.

## Conclusion

Our preliminary findings highlight the relevance of being able to attune to social information displayed in biological motions in a timely fashion during an ongoing task and being able to use that information to make attributions about feelings for understanding the social communication and interaction problems inherent in ASD. The research reported here extends our understanding of the relationship between various dimensions of social behavior (ToM, facial emotion recognition, social synchrony) that have not previously been fully explored. Namely, we found that spontaneous synchrony was related to ToM while intentional synchrony was related to clinical measures of attention and social responsiveness. Facial emotion recognition was not related to either ToM or social synchrony. Additional behavioral research with a larger sample size is needed to systematically evaluate static and dynamic measures of ToM, facial emotion recognition, and social synchrony in those with and without comorbid attention problems to be able to assess the importance of these findings. A further step will involve investigating whether any of these processes share underlying neural circuitry.

Overall, our findings suggest that methodologies that investigate the ‘process’ of social interactions unfolding hold much promise for providing measures with heightened resolution to better identify the essential qualities of social performance in naturalistic situations and isolate underlying mechanisms that may be disrupted in ASD. Such research holds promise for identifying the processing modules that can account for the full range of behaviors characteristic of ASD.

## Ethics Statement

The project was approved by the University of Massachusetts Medical School (Docket # H00001602) and Assumption College Institutional Review Boards (IRB # 2012-17, March 18, 2013). All parents gave written informed consent in accordance with the Declaration of Helsinki.

## Author Contributions

PF, JF, and RS study design, data collection, data analysis, data interpretation, and writing. TM data interpretation and writing. DC administering clinical assessments and data collection. CC data recruitment, data collection, and data entry.

## Conflict of Interest Statement

The authors declare that the research was conducted in the absence of any commercial or financial relationships that could be construed as a potential conflict of interest.
